# Fibrinogen consumption is related to intracranial clot burden in acute ischemic stroke: a retrospective hyperdense artery study

**DOI:** 10.1186/s12967-016-1006-6

**Published:** 2016-08-30

**Authors:** Slaven Pikija, Vladimir Trkulja, Johannes Sebastian Mutzenbach, Mark R. McCoy, Patricia Ganger, Johann Sellner

**Affiliations:** 1 Department of Neurology, Christian Doppler Medical Center, Paracelsus Medical University, Ignaz-Harrer-Str. 79, 5020 Salzburg, Austria; 2 Department for Pharmacology, School of Medicine, University of Zagreb, Zagreb, Croatia; 3 Division of Neuroradiology, Christian Doppler Medical Center, Paracelsus Medical University, Salzburg, Austria; 4 Department of Neurology, Klinikum rechts der Isar, Technische Universität München, München, Germany

**Keywords:** Fibrinogen, Clot burden, Ischemic stroke, Hyperdense artery, Computed tomography

## Abstract

**Background:**

Understanding the underlying mechanism of thrombus formation and its components is critical for effective prevention and treatment of ischemic stroke. The generation of thrombotic clots requires conversion of soluble fibrinogen to an insoluble fibrin network. Quantitative features of intracranial clots causing acute ischemic stroke can be studied on non-contrast enhanced CT (NECT). Here, we evaluated on-admission fibrinogen and clot burden in relation to stroke severity, final infarct volume and in-hospital mortality.

**Methods:**

We included 132 consecutive patients with ischemic stroke and presence of hyperdense artery sign admitted within 6 h from symptom onset. Radiological parameters including clot area (corresponding to clot burden) and final infarct volume were manually determined on NECT. National Institute of Health Stroke Scale (NIHSS) was used to quantify disease severity and short-term outcome.

**Results:**

Median patient age was 77, 58 % were women, and 63 % had an occlusion of the proximal middle cerebral artery segment. Thrombolysis was performed in 60 % and thrombectomy in 44 %. We identified several independent associations. Higher fibrinogen levels on admission were associated with smaller clot burden (p = 0.033) and lower NIHSS on admission (p = 0.022). Patients with lower fibrinogen had a higher clot burden (p = 0.028) and greater final infarct volume (p = 0.003). Higher fibrinogen was associated with a lower risk of in-hospital death or NIHSS score >15 if discharged alive (p = 0.028).

**Conclusions:**

Our study suggests that intracranial clot burden in acute ischemic stroke is associated with fibrinogen consumption, and shows a complex relationship with disease severity, infarct size and in-hospital survival.

**Electronic supplementary material:**

The online version of this article (doi:10.1186/s12967-016-1006-6) contains supplementary material, which is available to authorized users.

## Background

Cerebral blood flow can be interrupted by occlusion of major intracranial arteries and result in acute ischemic stroke [1]. Fibrinogen is a glycoprotein that helps in the formation of occluding blood clots. Fibrin, the product of thrombin’s proteolytic cleavage of fibrinogen, provides its biophysical and biochemical support [2]. Arterial thrombi are essentially composed of platelets with fibrin, whereas venous thrombi are rich of red-blood cells [3, 4]. Tissue-plasminogen activator (t-PA) is an thrombolytic agent for the treatment of acute ischemic stroke which dissolves fibrin bonds in the clot by activating plasminogen and is approved for iv treatment up to 4.5 h from symptom onset [5].

Several large prospective studied identified high fibrinogen plasma levels as an independent predictor of myocardial infarction and ischemic stroke [6–8]. While elevated fibrinogen is associated with other cardiovascular risk factors including age, smoking, blood pressure, and cholesterol, the relationship with stroke persisted even after correcting for these confounders [9]. Most recently, Potpara and coworkers identified the association of plasma fibrinogen with poor functional 30-day outcome in ischemic stroke [10]. Liu and coworkers studied fibrinogen levels in different stroke etiologies stratified according to the Trial of Org 10,172 in Acute Stroke Treatment (TOAST) classification [11]. While fibrinogen levels did not differ among the stroke subtypes, elevated d-dimer levels, a specific fibrinolysis marker, were typical for cardioembolic etiology.

Response to t-PA therapy and also efficacy of thrombectomy varies and presumably depends on a wide range of variables including location, time frame and radiological characteristics such as width, length and structure [12]. To this end, qualitative clot characteristics can be assessed on non-contrast enhanced CT (NECT). Fibrin is loosely packed in thrombi of cardioembolic origin and has better chances of recanalization using t-PA [4, 13]. Thrombi derived from large artery arteriosclerosis (LAA) comprise densely packed fibrin and are less likely to be resolved by medical strategies aimed at dissolving fibrin bonds.

An acute lowering of fibrinogen can be caused by degradation due to hyperreactive or stimulated systemic coagulation, resulting in increased thrombin formation and platelet activation [14]. Such rapid alterations of fibrinogen levels in the peripheral circulation are associated with clot burden in various acute thrombotic conditions. Fibrinogen consumption and a relationship with thrombin production has been reported for acute myocardial infarction, whereas this was not the case for stable coronary artery disease [15]. Notably, fibrinogen consumption is associated with a larger clot burden in pulmonary embolism [16]. Thus, it seems likely that fibrinogen degradation takes place in acute ischemic stroke caused by thrombotic occlusion of intracranial arteries. Here, we aimed to investigate the relationship between on-admission fibrinogen levels and radiological clot burden quantified within the first 6 h from symptom onset in acute ischemic stroke in relation with early clinical and radiological markers of outcome.

## Methods

### Study design

We reviewed medical records of consecutive patients admitted to the Christian Doppler Medical Center with acute ischemic stroke. The study period was January 2013 to January 2015. During the entire study period, there was no change of leading stroke staff, all three senior physicians were full-time neurologists.

**Fig. 1:**
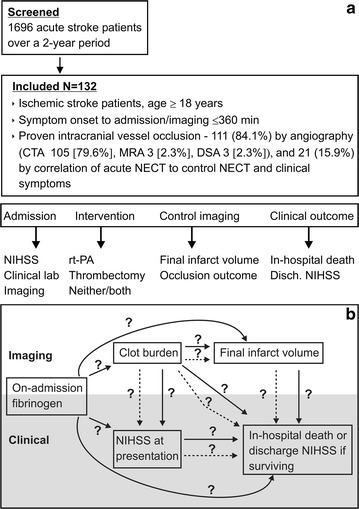
Study design. **a** Screening and inclusion of patients in the present analysis. **b** Diagram of the planned successive hypotheses about relationship between on-admission fibrinogen and imaging and clinical findings. *Full arrows* indicate direct associations and *dotted arrows* indicate the assumed indirect (mediated “through” mediator variables) associations. *CTA* angiography by computed tomography, *DSA* digital subtraction angiography, *MRA* angiography by magnetic resonance, *NECT* non-enhanced computed tomography, *NIHSS* National Institutes of Health Stroke Scale, *rt-PA* recombinant tissue plasminogen activator

The inclusion criteria were as follows: (a) age ≥18 years; (b) diagnosis of ischemic stroke; (c) proven intracranial vessel occlusion with quantifiable clot dimensions within 6 h after stroke onset (Fig. 1a); (d) presence of a hyperdense artery which was defined as “spontaneous visibility of complete or a part of” intracranial artery in segments with no calcifications [17]. We excluded cases without a hyperdense artery sign or non-ischemic intracerebral pathology was detected. For the purpose of group comparison we compiled a “non-MCA” group, which concerned all intracranial vessels other than branches of the middle cerebral artery.

### Ethics section

The protocol was in accordance with the ethical standards of our hospital’s committee for the protection of human subjects (protocol UN 2553). According to Austrian regulations, individualized informed consent is not required for routinely collected clinical and radiologic data as used in this study.

### Institutional standard procedure with acute stroke patients

Patients were treated according to the national stroke guidelines and local standard operation procedures for neuroimaging and mechanical thrombectomy. Minimal diagnostic work-up procedures included laboratory examinations on admission, extracranial Doppler und Duplex sonography of the brain-supplying arteries, monitoring at the stroke unit, extracranial transthoracic echocardiography, 24-h ECG monitoring and follow-up CT within 7 days. In-hospital variables were collected retrospectively for all patients via medical chart review and the IMPAX system (AGFA Healthcare, Mortsel, Belgium). Clinical disability on admission and transfer were routinely recorded with the National Institutes of Health Stroke Scale (NIHSS) by certified physicians.

### Quantification of the clot burden

NECT and CT angiography scans were performed in a multidetector CT scanner Sensation 64 (Siemens, Erlangen, Germany). The NECT scans were reconstructed into 4 mm thick adjacent slices through the entire brain. Two experienced neurologists blinded to the clinical information independently reviewed rated the scans. In case of disagreement, they discussed until a consensus was reached. The clot area was measured by delineating the hyperdense artery on NECT that corresponded to occlusion site on CT-A/MR-A/conventional angiography and/or matched with final infarct area. The region of interest was drawn around the hyperdense part of the artery and the area was automatically calculated using IMPAX software. When hyperdense artery area was seen on more than one slice the measured areas were summed [12]. In this regard, we used “clot area” (in mm^2^) as a measure of clot burden.

### Quantification of the final infarct volume

The follow-up CT scans were examined for infarct demarcation. The infarct area was manually delineated on each CT slice (4 mm height) which yielded area in cm^2^. Finally, the volume in cm^3^ was summed from the measured area and the corresponding slice thickness [18].

### Data analysis

Data analysis was conceived as a set of regressions aimed to test consecutive hypotheses about associations (“effects” used in the meaning of regression analysis, not necessarily implying causal relationship) between on-admission fibrinogen levels and co-incident or subsequent imaging and clinical findings (Fig. 1b). The analysis was driven by temporal and pathophysiological rationales: (a) the first step tested the association between on-admission fibrinogen and clot area (representing clot burden); (b) the next step tested the association between on-admission fibrinogen and clot area (simultaneously and separately) with NIHSS score at presentation. Differences in the strength of simultaneous and separate independent associations were to be considered an indication of possible direct and mediated (through the “effect” on clot area) “effects” of on-admission fibrinogen. In the same way, (c) the third step tested the association between on-admission fibrinogen and/or clot area and the final infarct volume; (d) the final step, following this concept, tested the association between on-admission fibrinogen and in-hospital clinical outcomes, accounting (simultaneously or separately) for clot area, final infarct volume and disease severity at presentation. For this purpose, a composite outcome of in-hospital death or survival but with NIHSS score >15 at discharge (moderate/severe or severe stroke) was analyzed. Continuous outcomes (clot area, infarct volume, NIHSS scores) were analyzed by fitting general linear models, whereas the composite of in-hospital mortality/NIHSS score at discharge >15 was analyzed by fitting modified Poisson regressions with robust error variance [19] to yield relative risks. Where required for achievement of normality of residuals, dependent and/or independent continuous variables were ln-transformed. All analyses were performed in SAS 9.3 for Windows (SAS Inc., Cary, NC).

## Results

### Patient characteristics and their relationship to on-admission fibrinogen levels

A total of 132 patients fulfilled the inclusion criteria. Most hyperdense artery signs could be confirmed by the performance of a CT-A (84.1 %) (Fig. 1a). Demographics and further characteristics of the cohort are shown in Table 1.

**Table 1:** Patient characteristics (N = 132)

Characteristic	Values
Demographics	
Age (years)	77 (19–97)
Men	55 (41.7)
Medical history	
Prior stroke/TIA	17 (12.9)
Atrial fibrillation	67 (50.8)
Peripheral artery disease	11 (8.3)
Carotid stenosis >50 %	15 (11.4)
Arterial hypertension	93 (70.5)
Diabetes mellitus	21 (15.9)
Chronic heart failure	18 (13.6)
Use of antiplatelets	41 (31.1)
Use of anticoagulants	12 (9.1)
Use of any antithrombotic	53 (40.2)
Stroke type by TOAST	
Cardioembolic	77 (58.3)
Unknown	37 (28.0)
Large artery atherosclerosis	15 (11.4)
Other	3 (2.3)
Clinical presentation	
NIHSS (points)	16 (0–32)
Serum glucose (mmol/L)	6.7 (3.1–12.9)
HbA1c (mmol/L)	5.5 (4.3-8.5)
Fibrinogen (μmol/L)	10.5 (3.1–24.3)
C-reactive protein (mg/L)	0.45 (0.01–21.2)
Acute treatment	
Thrombolysis (rt-PA)	79 (59.9)
Thrombectomy	58 (43.9)
Thrombectomy outcome (TICI)	
No perfusion (0)	10/58 (17.2)
Penetration, no distal filling (1)	3/58 (5.2)
Perfusion, <50 % distal filling (2a)	3/58 (5.2)
Inadequate (0–2a total)	16/58 (27.6)
Perfusion, >50 % distal filling (2b)	15/58 (25.9)
Full perfusion (3)	27/58 (46.5)
Adequate (2b–3 total)	42/58 (72.4)
Imaging particulars	
Symptoms to image (min)	116 (17–350)
Affected vessel	
Middle cerebral artery proximal	83 (62.9)
Middle cerebral artery distal	30 (26.6)
Basilar artery	8 (6.1)
Vertebral artery	6 (4.6)
Posterior cerebral artery	3 (2.3)
Anterior cerebral artery	1 (0.8)
Other vessel	1 (0.8)
Clot area (mm^2^)	25.2 (2.5–211)
Final infarct volume (mm^3^)	37.3 (0–518)
Control image finding	
Infarction	88 (66.7)
Hemorrhagic infarction	20 (15.2)
Resolution (infarct volume = 0)	11 (8.3)
None	13 (9.9)
Clinical outcome	
In-hospital mortality	26 (19.7)
NIHSS at discharge (points)	6 (0–30)

Data are median (range) or absolute numbers (percentage)

*HbA1c* glycated hemoglobin, *NIHSS* National Institutes of Health Stroke Scale, *rt-PA* recombinant human tissue plasminogen activator, *TIA* transitory ischemic attack, *TICI* thrombolysis in cerebral infarction grading, *TOAST* Trial of Org 10172 in Acute Stroke Treatment

On-admission fibrinogen ranged between 3.1 and 24.3 μmol/L and higher values were independently associated with older age (p = 0.002), higher C-reactive protein (p < 0.001), history of diabetes (p = 0.038) and history of heart failure (p = 0.020) (see Additional file 1: Table S1).

### Relationship between on-admission fibrinogen levels and clot area (clot burden)

Clot area ranged from 2.5 to 211 mm^2^ (Table 1). With adjustment for sex, history of carotid stenosis >50 % and type of the affected vessel (the only covariates with multivariate p < 0.1), higher on-admission fibrinogen was independently associated with a lower clot area (Table 2).

**Table 2:** Independent association between on-admission fibrinogen and clot burden represented by the clot area: summary of multivariate analysis

Independents	GMR (95 % CI)	p value
On-admission fibrinogen (by 2.718-fold)^a^	0.639 (0.424–0.964)	0.033
Men (vs. women)	1.453 (1.105–1.911)	0.008
History of carotid stenosis >50 %	1.485 (0.972–2.269)	0.068
Proximal vs. distal middle cerebral artery (MCA)	2.445 (1.770–3.376)	<0.001
Proximal MCA vs. “non-MCA” artery	1.535 (1.039–2.268)	0.038

Ln-transformed clot area values were analyzed and results are presented as geometric means ratio (GMR) with 95 % confidence intervals by unit or level change in an independent analysis

The initial general linear model fitted to ln(clot area) included all independents selected from the variables depicted in Table 1 (except for symptom severity on-admission [NIHSS], acute treatment, final infarct volume, control image finding and clinical outcome) based on a trend towards univariate association with this outcome (p < 0.1) [ln(fibrinogen), sex, history of carotid stenosis, affected blood vessel (proximal or distal middle cerebral artery, or “non-middle cerebral artery”), age and prior use of antiplatelets]. Variables from this full model were then successively removed (age p = 0.562, prior antiplatelet use p = 0.115) in the order of the highest p value, if p > 0.100 (backward elimination). Two-term interactions between ln(fibrinogen) and each of the other effects remaining in the model were tested, but were insignificant with p > 0.500 and excluded. The final model is shown

^a^Since on-admission fibrinogen was ln-transformed (to achieve normality of residuals), the “effect” of on-admission fibrinogen is presented as GMR by 2.718-fold increase

### Relationship between on-admission fibrinogen levels, clot area (clot burden) and severity of symptoms at presentation

**Fig. 2:**
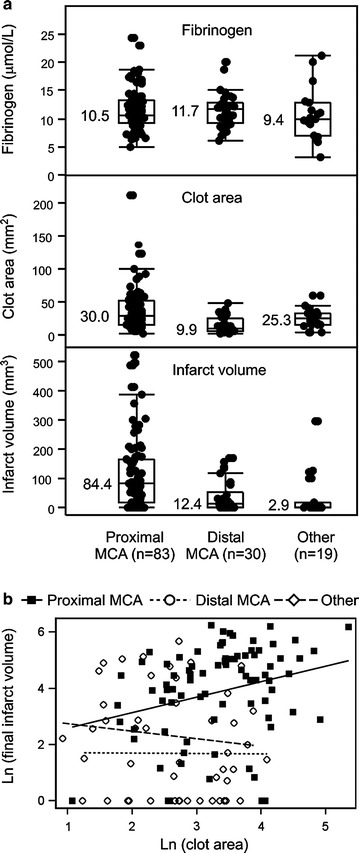
Relationship between on-admission fibrinogen, type of the affected vessel, clot area (clot burden) and final infarct volume. **a** On-admission fibrinogen (μmol/L) (*upper panel*), clot areas (mm^2^) (*middle panel*) and final infarct volumes (mm^3^) according to the type of the affected vessel. *Dots* are individual values, *horizontal lines* are medians (numerical values depicted), *boxes* indicate upper and lower quartiles and *bars* are inner fences [median ± (1.5 × interquartile range)]. Values outside fences are outliers. **b** Fitted (adjusted) regression of ln(infarct volume) on ln(clot area) by vessel type, from the model depicted in Table 4 in the main text. *MCA* middle cerebral artery

NIHSS scores at presentation ranged from 0 to 32 points (Table 1). With adjustment for time elapsed since symptom onset to imaging, age, C-reactive protein and serum glucose levels, type of the affected vessel and clot area (the only covariates with multivariate p < 0.1) higher on-admission fibrinogen was independently associated with lower NIHSS scores (Table 3, Model 1). Higher clot area was associated with higher NIHSS scores but with borderline statistical significance when fibrinogen was in the model (p = 0.054; Table 3, Model 1). In separate models (with all other effects) including either fibrinogen (Table 3, Model 2) or clot area (Table 3, Model 3), each were independently associated with more severe symptoms at presentation. Following independent associations were evaluated: on-admission fibrinogen—clot area; on-admission fibrinogen—symptom severity at presentation; clot area—symptom severity at presentation, attenuation of “effects” of fibrinogen and clot area on symptom severity when both were accounted for the assumption that the “effect” of on-admission fibrinogen on symptom severity is at least in part mediated through its “effect” on the clot area (Fig. 2).

**Table 3:** Independent association of on-admission fibrinogen and clot burden represented by the clot area with severity of symptoms at presentation (NIHSS): summary of multivariate analysis

Independents	GMR (95 % CI)	p value
Model 1		
On-admission fibrinogen (by 2.718-fold)^a^	0.683 (0.473–0.987)	0.042
Clot area (by 10 mm^2^)	1.039 (0.999–1.081)	0.054
Symptom onset to image (by 10 min)	0.984 (0.972–0.997)	0.023
Age (by 5 years)	1.507 (1.010–1.093)	0.014
C-reactive protein (by 1 mg/L)	1.092 (1.045–1.142)	<0.001
Serum glucose (by 1 mmol/L)	1.064 (1.007–1.124)	0.027
Proximal vs. distal middle cerebral artery (MCA)	1.454 (1.110–1.904)	0.007
Proximal MCA vs. “non-MCA” artery	1.866 (1.355–2.570)	<0.001
Model 2 (clot area not included; shows just fibrinogen—all other effects similar as in Model 1)		
On-admission fibrinogen (by 2.718-fold)^a^	0.649 (0.449–0.938)	0.022
Model 3 (fibrinogen not included; shows just clot area—all other effects similar as in Model 1)		
Clot area (by 10 mm^2^)	1.045 (1.005–1.087)	0.028

Ln-transformed NIHSS scores were analyzed and results are presented as geometric means ratio (GMR) with 95 % confidence intervals by unit or level change in an independent

All variables depicted in Table 1 (except for acute treatment, control image finding, final infarct volume and clinical outcome) were tested for at least a trend (p < 0.1) of univariate association with the NIHSS score at presentation. Ln(fibrinogen), clot area, time since symptom onset to imaging (surrogate for admission), age, C-reactive protein, type of the vessel affected, serum glucose and history of heart failure met this criterion, but the last variable was removed from the final model due to p > 0.5. Three models were fitted differing regarding inclusion of both ln(fibrinogen) and clot area (Model 1), or just ln(fibrinogen) (Model 2) or just clot area (Model 3) along with other effects. The interaction terms between ln(fibrinogen) or clot areal and vessel type were highly insignificant

*NIHSS* National Institutes of Health Stroke Severity scale

^a^Since on-admission fibrinogen was ln-transformed (to achieve normality of residuals), the “effect” of on-admission fibrinogen is presented as GMR by 2.718-fold increase

### Relationship between on-admission fibrinogen, clot burden and final infarct volume

The relationship between on-admission fibrinogen, clot area and final infarct volume appeared complex and conditional on the affected vessel (Fig. 1 depicts individual values by type of the affected vessel). With adjustment for C-reactive protein and glucose levels, performed thrombectomy [options: not done, done but inadequate perfusion (TICI grade 0–2a) or adequate (TICI grade 2b–3)] and type of the affected vessel (the only covariates with multivariate p < 0.1), higher on-admission fibrinogen was independently associated with a lower infarct volume (Table 4). In contrast, larger clot area was associated with a higher infarct volume, but only in the case of proximal MCA (p = 0.069 for the clot area*vessel type interaction) (Table 4) [Fig. 2 depicts adjusted regressions of ln(infarct volume) on ln(clot area) by vessel type]. The association between on-admission fibrinogen and infarct volume was unchanged when the clot area was removed, and the association between clot area and infarct volume remained unchanged when fibrinogen was removed from the model (not shown).

**Table 4:** Independent association of on-admission fibrinogen and clot burden represented by the clot area with final infarct volume: summary of multivariate analysis

Independents	GMR (95 % CI)	p value
On-admission fibrinogen (by 2.718-fold)^a^	0.221 (0.081–0.601)	0.003
Clot area (by 2.718-fold)^a^		
If proximal middle cerebral artery (MCA) affected	1.712 (1.096–2.676)	0.018
If distal MCA affected	0.759 (0.354–1.625)	0.475
If “non-MCA” artery affected	0.612 (0.316–1.707)	0.346
C-reactive protein (by 1 mg/L)	1.275 (1.121–1.451)	<0.001
Serum glucose (by 1 mmol/L)	1.173 (1.011–1.362)	0.036
Thrombectomy with TICI 2b–3 vs. no thrombectomy	0.377 (0.191–0.743)	0.005
Thrombectomy with TICI 2b–3 vs. TICI 0–2a	0.513 (0.193–1.363)	0.179
Proximal vs. distal MCA	6.001 (2.501–14.4)	<0.001
Proximal MCA vs. “non-MCA” artery	15.4 (6.27–37.7)	<0.001

Ln-transformed infarct volume values were analyzed and results are presented as geometric means ratio (GMR) with 95 % confidence intervals by unit or level change in an independent

All variables depicted in Table 1 [except for severity of clinical symptoms at presentation (National Institutes of Health Stroke Scale score), control image finding and clinical outcome] were tested for at least a trend (p < 0.1) of univariate association with the final infarct volume and were included in the model on this criterion. Two-term interactions between on-admission fibrinogen and vessel type or treatment with thrombectomy, as well as between clot area and thrombectomy were insignificant (p > 0.500) and excluded, whereas clot area*vessel type interaction was significant at alpha 0.1 (p = 0.069) and the inclusive model had the best fit (Akaike’s information criterion 505.5, Bayesian information criterion 508.3) and is depicted

*TICI* thrombolysis in cerebral infarction grading

^a^Since on-admission fibrinogen and clot area were ln-transformed (to achieve normality of residuals), the “effects” are presented as GMRs by 2.718-fold increase

### Relationship between on-admission fibrinogen, clot burden, symptom severity at presentation, final infarct volume and clinical outcomes—in-hospital mortality and symptom severity in hospital survivors

A total of 26 patients (19.7 %) died during the hospital stay (Table 1). NIHSS score at discharge in survivors (n = 106) varied between 0 and 30 (Table 1) and was >15 (moderate/severe or severe stroke) in 27 (25.5 %) of them. Overall, 53 (40.2 %) patients either died in hospital or were discharged with NIHSS score >15.

We found that higher on-admission fibrinogen was associated with a lower risk of in-hospital death/NIHSS score at discharge >15 (Table 5, Model 1). This was confirmed after adjustment for age, sex, time since symptom onset to imaging, C-reactive protein and glucose levels and type of the affected vessel (covariates found independently associated with on-admission fibrinogen or clot area or final infarct volume or NIHSS score at presentation, Tables 1, 2, 3, 4), and thrombectomy.

**Table 5:** Association of on-admission fibrinogen, clot burden represented by clot area, final infarct volume and symptom severity at presentation (NIHSS score) with the risk of in-hospital death or survival with NIHSS score at discharge >15: summary of multivariate analysis

Independents	RR (95 % CI)	p value
Models 1–4: variable of interest + default adjustments^a^		
Model 1—variable of interest: on-admission fibrinogen		
On-admission fibrinogen (by 2.718 fold)^b^	0.478 (0.247–0.924)	0.028
Model 2—variable of interest: clot area		
Clot area (by 10 mm^2^)	1.057 (1.013–1.104)	0.010
Model 3—variable of interest: final infarct volume		
Final infarct volume (by 10 mm^3^)	1.030 (1.019–1.043)	<0.001
Model 4—variable of interest: NIHSS at presentation		
NIHSS at presentation (by 1 score point)	1.097 (1.071–1.123)	<0.001
Model 5—full model: all variables of interest + adjustments		
On-admission fibrinogen (by 2.718-fold)^b^	0.790 (0.445–1.401)	0.420
Clot area (by 10 mm^2^)	0.990 (0.942–1.031)	0.559
Final infarct volume (by 10 mm^3^)	1.015 (0.998–1.032)	0.084
NIHSS at presentation (by 1 score point)	1.087 (1.060–1.114)	<0.001
Age (by 5 years)	1.065 (0.965–1.176)	0.208
Male gender	1.205 (0.815–1.784)	0.353
Symptom onset to imaging (admission) (by 10 min)	0.997 (0.973–1.021)	0.420
C-reactive protein (by 1 mg/L)	1.032 (0.982–1.084)	0.211
Glucose (by 1 mmol/L)	1.151 (1.041–1.273)	0.006
Proximal vs. distal middle cerebral artery (MCA)	0.397 (0.135–1.145)	0.087
Proximal MCA vs. “non-MCA” artery	2.259 (1.136–4.491)	0.020
Thrombectomy with TICI 2b–3 vs. no thrombectomy	0.700 (0.404–1.213)	0.204
Thrombectomy with TICI 2b–3 vs. TICI 0–2a	0.484 (0.268–0.875)	0.016

Data are presented as relative risks (RR) with 95 % confidence intervals

*NIHSS* National Institutes of Health Stroke Severity scale

*TICI* thrombolysis in cerebral infarction grading

^a^Models 1–4 each consisted of a variable of primary interest and a set of default adjustments based on their independent associations with the variables of primary interest (Tables 1, 2, 3, 4): age, gender, time elapsed since symptom onset to imaging (reflects admission), C-reactive protein and glucose levels on admission, affected vessel (proximal or distal middle cerebral artery or “non-MCA” artery) and performed thrombectomy (none, with perfusion TICI grade 0–2a or grade 2b–3). Model 5 included all variables of primary interest and all adjustments

^b^Since on-admission fibrinogen was ln-transformed (as in all previous models), the “effects” are presented as GMRs by 2.718-fold increase

With the same adjustments, higher clot area (Table 5, Model 2), larger infarct volume (Table 5, Model 3) and higher NIHSS at presentation (Table 5, Model 4) were each associated with a higher risk of in-hospital death/NIHSS score at discharge >15.

In a full model (Table 5, Model 5), i.e., with all “default adjustments” and including fibrinogen, clot area, infarct volume and NIHSS at presentation—only higher NIHSS at presentation (p < 0.001) remained independently associated with an increased risk.

The sequence of independent associations between on-admission fibrinogen and clot area, fibrinogen and clot area with NIHSS score at presentation, fibrinogen and clot area with final infarct volume and associations depicted in Table 5. Together with the attenuation of the “effects” of on-admission fibrinogen, clot area and infarct volume on the clinical outcome when all (together with NIHSS at presentation) were in the same model, implicates that the association between on-admission fibrinogen and assessed clinical outcomes is mediated through its association with clot area, infarct volume and severity of disease at presentation.

## Discussion

Understanding the underlying mechanism of thrombus formation and its consequences is critical for effective prevention and treatment of ischemic stroke. This study disclosed an independent inverse relation between on-admission fibrinogen levels and clot burden. This finding points at in vivo fibrinogen consumption in and after the process of thrombus formation. Moreover, fibrinogen degradation and clot size showed a complex relationship with disease severity, infarct size and in-hospital survival.

Fibrinogen is a central molecule in thrombosis and hemostasis and implicated in additional conditions including as well as in pathologies including inflammation, host defense, cancer, and neuropathology. Indeed, elevated fibrinogen is one of the most prevalent risk factors for thrombotic disorders [20–22]. We corroborate the reported independent associations between higher on-admission fibrinogen levels and older age, higher C-reactive protein, diabetes and history of cardiovascular disease in acute ischemic stroke patients. Moreover, higher clot burden is associated with more severe stroke symptoms at presentation [18, 19]. We expand these observations on the basis of our second analysis step by reporting an independent association between lower fibrinogen and more severe presenting symptoms, and between higher clot burden and disease severity. In the full model (Table 3) both associations weakened, and the latter one reached “only” a borderline statistical significance. Since the first-step analysis demonstrated the association between the two, this phenomenon was expected and indicated that, at least in part, the “link” between on-admission fibrinogen levels and symptoms at presentation “went through” its effect on the clot burden. Moreover, the fact that the strength of association between fibrinogen levels and symptom severity was less reduced than the strength of association between the clot burden and symptom severity suggests that fibrinogen might reflect clot perturbations on a finer scale (thrombus formation/lysis) and therefore could be a better indicator of the extent of thrombus, while clot burden measurement is essentially flawed by imperfect methodology. We propose that NECT depicts only a part or only the erythrocyte-rich part of the thrombus whilst the platelet and/or fibrin-rich (and thus hypoattenuating) parts are not visible on NECT. It also needs to be taken into account that intracranial clots are not homogenous and ongoing apposition and endogenous thrombolysis takes place [23, 24]. This assumption is backed by findings in ischemic heart disease, where proximal and distal of the fibrin-thrombocyte rich nidus develop after the local plaque rupture in coronary vessels [25]. Local hemodynamics and collaterals may also contribute to qualitative and quantitative alterations of the clot [23, 26].

The final infarct volume is a consequence of a multitude of factors or their combinations, such as the presence of collaterals or the choice of treatment, and could serve as the stroke outcome surrogate [27]. At the third step, the present analysis indicated that both on-admission fibrinogen (inversely) and clot burden were independently associated with the final infarct volume. However, the latter association was conditional on the type of the affected vessel—it was only relevant in occlusion of the middle cerebral artery.

Finally, the most complex relationship observed was the relation of on-admission fibrinogen and poor in-hospital outcome (death or NIHSS score at discharge >15). This points at an independent association between higher fibrinogen and a reduced risk—but not when “intermediate” outcomes (clot area, final infarct volume, symptom severity at presentation) to which fibrinogen was also related, were accounted for, suggesting that the “effect” of fibrinogen was conveyed “through” these mediators. Although such a sequence of events appears mechanistically plausible, the present observations should be taken with a caution since we considered only the on-admission fibrinogen levels. Fibrinogen levels steadily rise over 120 h after stroke, are linked to a poor outcome and are decreases with t-PA treatment and subsequently raise risk for intracranial hemorrhage [28, 29]. Of note, Sun and coworkers found that the decrease in fibrinogen less than 2 g/L multiplies the odds of early parenchymal hemorrhage as a complication of intravenous thrombolysis by factor 12.8 [30].

The present study has a several limitations, which need to be considered in future studies. The retrospective studies might have introduced bias and we did not assess plasma levels of tissue plasminogen activator or plasmin activator inhibitor-1 as well as fibrinogen degradation products. The major limitation to generalizability, however, arises from the fact that we studied only patients with hyperdense artery signs. Up to 70 % of occlusive thrombi on NECT are hyperdense, but there are patients with the major vessel occlusion without a hyperdense artery and they were not present in our study. Additionally, small vessel occlusion i.e. lacunar strokes were not included as clot area measurement in non-hyperdense thrombi is not plausible. Overall, while we used a timely methodology, we admit that smaller hyperdense signs may have been missed. On the other hand, the study has several strengths—all patients underwent standardized diagnostic and therapeutic procedures, medical history data were complete, all radiological assessments were done after pre-defined criteria by raters blinded to clinical outcomes and data were viewed in a sensible and thorough way.

## Conclusion

We investigated the relationship between on-admission fibrinogen levels and clot burden, symptom severity at presentation, and in-hospital clinical and radiological outcomes in a moderately sized sample of highly selective stroke patients with the sign of acute vessel occlusion within the first 6 h after the stroke onset. Importantly, plasma fibrinogen could predict the majority of clinical and radiological outcomes. The results are novel and provide an important impulse to further unravel dysregulation of coagulation pathways in acute ischemic stroke.

## Additional file

10.1186/s12967-016-1006-6 Baseline patient characteristics associated with on-admission fibrinogen levels: summary of multivariate analysis.

## Abbreviations

CT: computed tomography
MRI: magnetic resonance imaging
NECT: non-contrast enhanced CT
NIHSS: National Institute of Health Stroke Scale
TOAST: Trial of Org 10172 in Acute Stroke Treatment
t-PA: tissue-plasminogen activator
CT-A: computed tomography-angiography
MR-A: magnetic resonance imaging-angiography
TICI: thrombolysis in cerebral infarction
