# Supplementary material for: Fibrinogen consumption is related to intracranial clot burden in acute ischemic stroke: a retrospective hyperdense artery study
Source: J Transl Med. 2016 Aug 30;14(1):250. doi: 10.1186/s12967-016-1006-6 (PMC5006507; doi:10.1186/s12967-016-1006-6)
Supplement: Supplementary file 1 — 10.1186/s12967-016-1006-6 Baseline patient characteristics associated with on-admission fibrinogen levels: summary of multivariate analysis. [file 12967_2016_1006_MOESM1_ESM.docx]

**Table S1**. **Baseline patient characteristics associated with on-admission fibrinogen levels: summary of multivariate analysis.** Ln-transformed fibrinogen levels were analyzed and results are presented as geometric means ratios (GMR) with 95% confidence intervals.

| Independents |  | GMR (95% CI) |  | p-value |
| --- | --- | --- | --- | --- |
| Age (by 5 years) |  | 1.039 (1.011-1.047) |  | 0.002 |
| C-reactive protein (by 1 mg/L) |  | 1.039 (1.019-1.060) |  | <0.001 |
| History of diabetes mellitus |  | 1.154 (1.008-1.321) |  | 0.038 |
| History of chronic heart failure |  | 1.200 (1.029-1.399) |  | 0.020 |
| Affected is MCA (vs. other) |  | 1.143 (0.988-1.322) |  | 0.072 |

The initial general linear model fitted to ln(fibrinogen) included age, sex, medical history (history of cerebrovascular incidents, peripheral artery disease, atrial fibrillation, diabetes, hypertension, carotid occlusion >50%, chronic heart failure; as well as prior use of antiplatelets or anticoagulants), timing between symptom onset and admission/imaging and on-admission laboratory (C-reactive protein, serum glucose and HbA1c) and stroke (cardioembolic or “other”, affected is middle cerebral artery [MCA] or “other”) characteristics as independents. Effects were then removed in the order of the highest p-value (backward elimination) if p>0.100. The final model is shown.
